# *cis*MEP: an integrated repository of genomic epigenetic profiles and *cis*-regulatory modules in Drosophila

**DOI:** 10.1186/1752-0509-8-S4-S8

**Published:** 2014-12-08

**Authors:** Tzu-Hsien Yang, Chung-Ching Wang, Po-Cheng Hung, Wei-Sheng Wu

**Affiliations:** 1 Department of Electrical Engineering, National Cheng Kung University, Tainan, Taiwan

**Keywords:** epigenetic profile, *cis*-regulatory module, chromatin regulation, histone modification, sequence conservation, regulatory protein

## Abstract

**Background:**

*Cis*-regulatory modules (CRMs), or the DNA sequences required for regulating gene expression, play the central role in biological researches on transcriptional regulation in metazoan species. Nowadays, the systematic understanding of CRMs still mainly resorts to computational methods due to the time-consuming and small-scale nature of experimental methods. But the accuracy and reliability of different CRM prediction tools are still unclear. Without comparative cross-analysis of the results and combinatorial consideration with extra experimental information, there is no easy way to assess the confidence of the predicted CRMs. This limits the genome-wide understanding of CRMs.

**Description:**

It is known that transcription factor binding and epigenetic profiles tend to determine functions of CRMs in gene transcriptional regulation. Thus integration of the genome-wide epigenetic profiles with systematically predicted CRMs can greatly help researchers evaluate and decipher the prediction confidence and possible transcriptional regulatory functions of these potential CRMs. However, these data are still fragmentary in the literatures. Here we performed the computational genome-wide screening for potential CRMs using different prediction tools and constructed the pioneer database, *cis*MEP (*cis*-regulatory module epigenetic profile database), to integrate these computationally identified CRMs with genomic epigenetic profile data. *cis*MEP collects the literature-curated TFBS location data and nine genres of epigenetic data for assessing the confidence of these potential CRMs and deciphering the possible CRM functionality.

**Conclusions:**

*cis*MEP aims to provide a user-friendly interface for researchers to assess the confidence of different potential CRMs and to understand the functions of CRMs through experimentally-identified epigenetic profiles. The deposited potential CRMs and experimental epigenetic profiles for confidence assessment provide experimentally testable hypotheses for the molecular mechanisms of metazoan gene regulation. We believe that the information deposited in *cis*MEP will greatly facilitate the comparative usage of different CRM prediction tools and will help biologists to study the modular regulatory mechanisms between different TFs and their target genes.

## Background

Differential gene expression distinguishes distinct cell types in the differentiation of cells [1]. Correct temporal and spatial control of gene expression is crucial in different developmental stages in metazoans. In metazoan cells, transcriptional regulation of gene expression is controlled in a modular manner by specific DNA sequences located in the intergenic regions or in the introns [2]. Identifying these regulatory DNA sequences, or the *cis*-regulatory modules (CRMs), and their functions in gene transcriptional regulation can both expand our understanding of differential gene regulation and have potential application in medicine as well.

Three major CRM functions are known. In metazoan cells, CRMs can function as promoters, enhancers/silencers or insulators [3]. Promoter regulatory DNA sequences direct RNA polymerase to initiate gene transcription [4]. Enhancer/silencer CRMs operationally activate/repress the transcriptional expression of target genes with the help of regulatory proteins bound to them [5]. Finally, insulators block the effect of other enhancers with proteins such as CTCF [6]. Two major factors are closely related to CRM functionality: transcription factor (TF) binding and the shared chromatin epigenetic profiles [3,7-9]. Depending on the epigenetic profiles the regulatory sequences bear, different TFs and regulatory proteins are recruited to CRMs, hence determining the functions of them [7,10]. For example, it has been reported that in promoter regions the RNA polymerase II signals can be identified and in enhancer regions H3K4me and nucleosome depletion can be observed [11].

While experimental approaches allow the determination of CRMs, genome-wide systematic identification of instances of CRMs nowadays still mainly resorts to computational methods due to the time-consuming, small-scale and low-throughput nature of experimental approaches [12-14]. The existing CRM searching computational strategies in *Drosophila *are based on two features of CRMs, namely the transcription factor binding sites (TFBS) and phylogeny. Conserved homologous and non-coding DNA sequences between related species are thought to be closely related to gene regulation [3]. Some CRM prediction algorithms used the window clustering method or the probabilistic modelling to find regulatory sequences with significant clustering of high densities of TFBSs [15,16]. Others utilized the phylogenetic footprinting on comparative homologous sequences between related species to reveal potential CRMs [3,13]. And many methods are hybrids of the two strategies. Various CRM prediction tools have been developed based on these CRM searching strategies. But the accuracy and reliability of different CRM prediction tools are still unclear. Without comparative cross-analysis of different prediction results and combinatorial consideration with extra experimental information, there is no easy way to assess the confidence of the predicted CRMs. And to use the CRM prediction tools, it requires the users to get fully familiar with these tools and data preprocessing. This further prohibits the comprehensive and comparative usage of these CRM computational methods.

To fill this gap, there is an urgent need to perform the genome-wide CRM screening using different prediction tools for comparative referencing and to manually collect experimental information for assessing the confidence of these potential CRMs. Since most of the prediction algorithms did not rely on the epigenetic features of CRMs, the epigenetic profiles provide excellent resources for assessing the confidence of these prediction results. In *Drosophila*, there are deposited high-throughput data identifying the epigenetic marks [11]. But the information is still fragmentary in different resources and there is no easy way for combinational visualization of the predicted CRMs and the epigenetic profiles thus far. Hence we performed the genome-wide screening for CRMs using different prediction tools and constructed the pioneer database, *cis*MEP (*cis*-regulatory module epigenetic profile database), to deposit the CRM prediction results and to integrate these computationally identified CRMs with manually collected genome-wide experimental epigenetic marks as the confidence assessment resources. Researchers can further assess the CRM prediction confidence by the implemented filters based on the potential regulatory functional annotation or combinations of histone modifications. In addition to the deposited potential CRMs, we also retrieved experimentally verified CRMs from the REDfly database [12] and other literature to distinguish putative and known CRMs. *cis*MEP aims to provide a user-friendly interface for researchers to understand and decipher the confidence and possible functions of potential CRMs through their epigenetic profiles. We believe that this will facilitate biologists to design subsequent experiments for gene regulatory analysis. *cis*MEP is available online at http://cosbi3.ee.ncku.edu.tw/cisMEP/.

## Construction and contents

### Collection of systematic computational CRM screening results and construction of *cis*MEP

**Figure 1:**
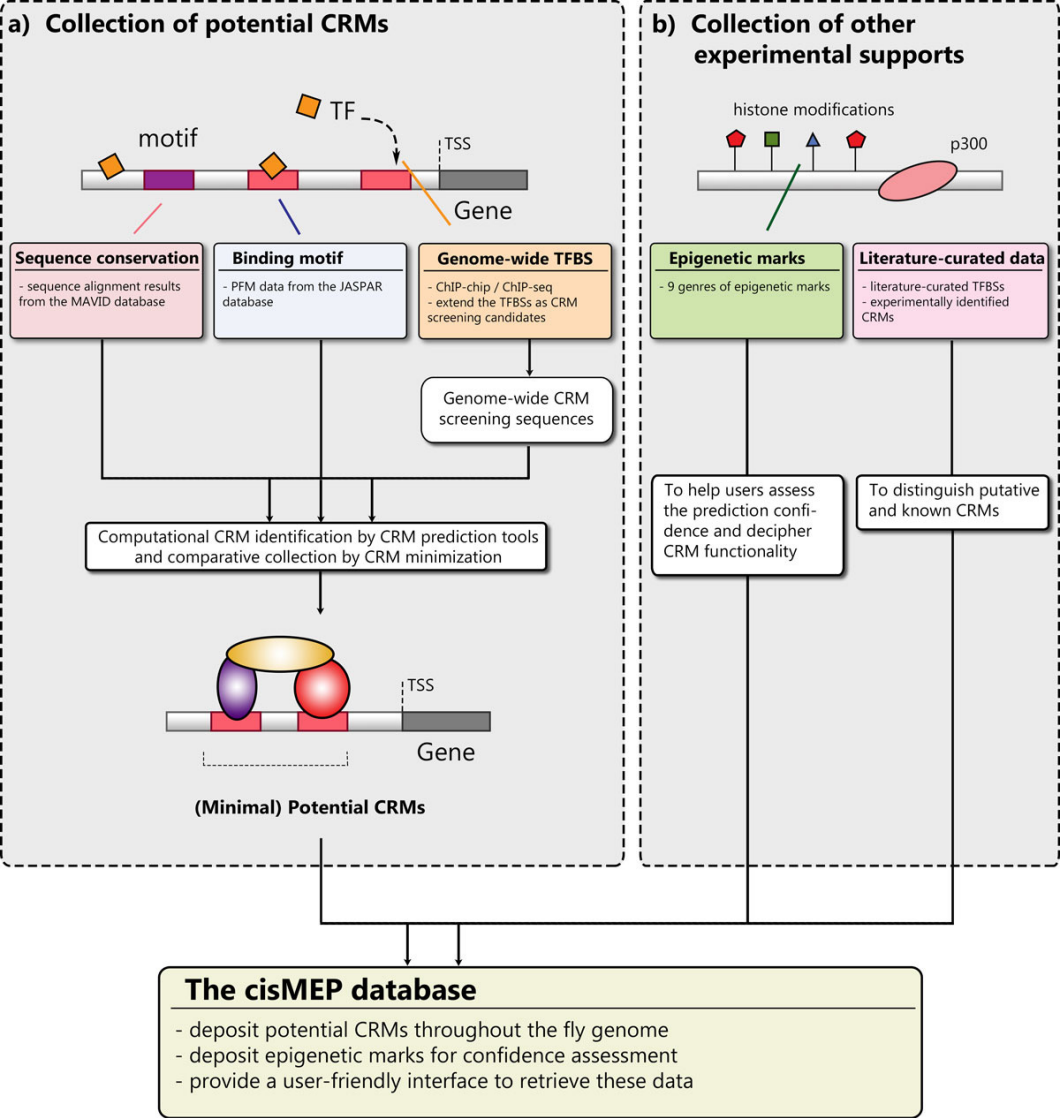
**Construction of the *cis*MEP database**. *cis*MEP gathered the computationally identified CRMs and collected the literature-curated TFBS location data and nine genres of epigenetic data. (a) We have performed different CRM prediction algorithms and gathered these identified potential CRMs for comparative cross-referencing. (b) The genomic epigenetic marks are mapped to the locations of these potential CRMs for prediction confidence assessment and potential CRM function determination. We further retrieved the literature-curated CRMs to distinguish putative and known CRMs.

We used currently available computational CRM prediction tools to perform and to collect genome-wide systematic screening for potential CRMs and gathered different experimentally-identified epigenetic profiles as confidence assessment and functional deciphering resources. For CRM identification, we adopted the minimal sequence definition proposed by the REDfly database for the comparative collection of potential CRMs identified by different CRM screening tools. We further retrieved experimentally verified CRMs from the REDfly database and other literatures to distinguish putative and known CRMs. To provide an easy-to-use interface for integrative viewing and retrieving these data, we constructed the *cis*MEP database to deposit the information. The construction of *cis*MEP is sketched in Figure 1 and details of the collection and statistics of experimental epigenetic data can be found in the following sections. The collection of potential CRMs are stated as follows.

To perform the systematic genome-wide CRM screening, we selected five CRM prediction tools which have been verified for *Drosophila *and are still publicly available [13]: *cis*Module [17], *cis*PlusFinder [18], ClusterBuster [16], MCAST [15] and MultiModule [19]. These methods were based on TFBS clustering or comparative phylogeny. For MCAST and ClusterBuster, statistic models constructed from the features of TFBS clustering are used to search for significant regulatory sequences. In these two methods, the transcription factor binding motifs are required and should be represented in the PFM format. For *cis*PlusFinder and MultiModule, CRM prediction is based on sequence conservation across multiple species. And in *cis*Module, both the binding motifs and the sequence conservation under purifying selection are utilized in a hidden Markov model. Different input data and data preprocessing are required for running all these tools (Figure 1-a).

CRMs carry out their functions by recruiting different regulatory proteins to regulatory motifs within the CRM sequences. Hence to generate candidate sequences for genome-wide CRM screening, we collected the genome-wide TFBS data from the modENCODE project [20]. 39,339 TFBSs identified by the ChIP-chip or ChIP-seq techniques were used to generate the candidate sequences for screening genome-wide potential CRMs. A candidate sequence for CRM searching is defined by the TFBS sequence and extra 3 kb sequences extended from upstream and downstream of the TFBS respectively. The choice of extra 6 kbp is due to the fact that the sequence lengths of experimentally identified CRMs range from a few hundred base-pairs to a few thousand base-pairs long and the default sliding window in most prediction tools are generally 200-1000 base-pairs [13]. To generate the prediction results, we retrieved the genome sequence information of *Drosophila melanogaster *from Flybase (Version 5.51) [21], the sequence alignment results of *Drosophila melanogaster, Drosophila yakuba, Drosophila ananassae, Drosophila virilis *and *Drosophila pseudoobscura *from the MAVID multiple alignment database [22] and the PFM data of TFBS motifs from the JASPAR database [23]. Default parameter settings were used in these prediction tools and the time-consuming genome-wide CRM screening using different available CRM prediction tools was made possible with the assistance of parallel computing techniques.

**Figure 2:**
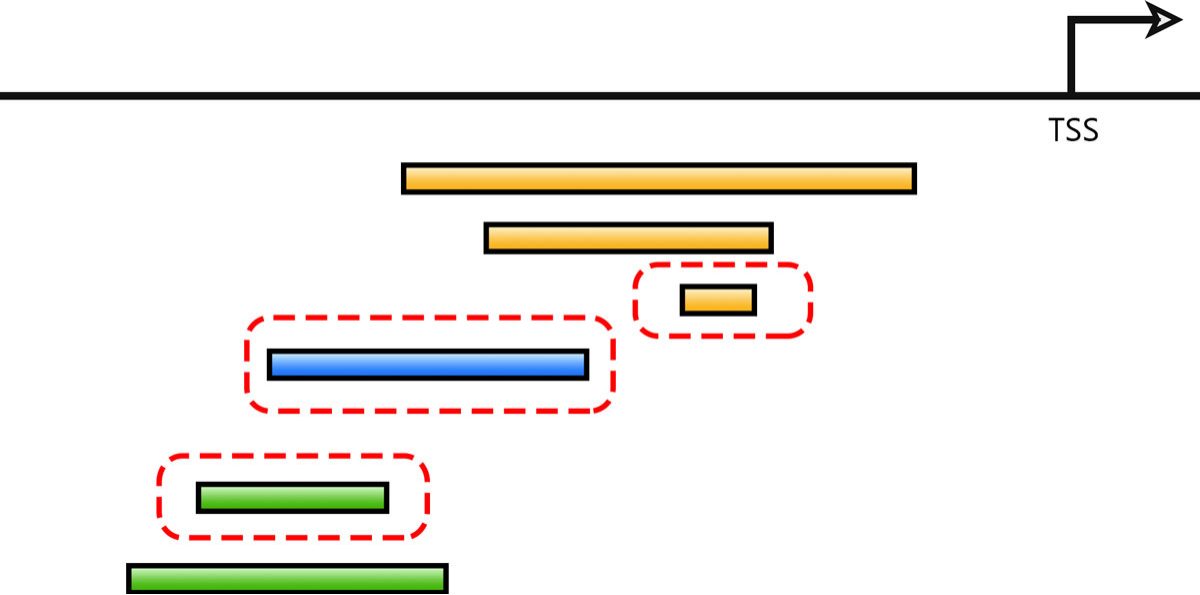
**The sketch plot for minimizing overlapping CRMs**. The potential CRMs predicted by different CRM screening tools were grouped into nested sets and the minimal sequences in the nested sets were selected as the representing CRMs. In the figure, CRMs forming a nested set are in the same colour and the minimal representing CRM is circumscribed with a dash frame.

Different computational CRM searching algorithms may provide different potential CRMs. A predicted CRM is of higher confidence if more CRM screening algorithms find it to be a potential CRM. Hence for comparative referencing, we adopted the minimal sequence definition proposed by the REDfly database. For a group of overlapping potential CRMs, we classified these CRMs into nested sets and took the minimal sequences in the nested sets as the representing CRMs (See Figure 2). In total, there are 410,364 (minimal) potential CRMs deposited in *cis*MEP. For these potential CRMs, we further distinguish putative and known CRMs from them based on the collected experimentally verified CRMs (Figure 1-b). Finally, we related a potential CRM to those genes regulated by the literature-curated TFBSs or the literature-curated CRMs residing within the range of this CRM, if available. We further linked the CRM annotation to the gene annotation by finding those genes within 100kbp upstream or downstream the potential CRMs. The linkage information of the CRM annotation and the gene annotation can be visualized in the feature of "Transcriptional Regulation Inference Map" in the detailed CRM information page.

### Collection and statistics of experimentally identified TFBSs and epigenetic data

To provide confidence assessment for the potential CRMs in *cis*MEP (Figure 1-b), we collected the literature-curated transcription factor binding site (TFBS) data and nine genres of epigenetic data, including two deduced chromatin state models, chromatin accessibility, cross-species sequence conservation, experimentally identified promoter regions, polycomb group target regions, annotated regulatory elements, histone variant nucleosomes, RNA polymerase II binding and pausing sites, and 40 kinds of histone modifications. The data statistics are stated as follows.

#### Literature-curated CRMs and TFBSs

Experimentally identified CRMs were retrieved from the REDfly v3.0 database [12] and CAD [24] (CRM Activity Database). REDfly collected experimentally verified CRMs in the literature that were unambiguously demonstrated to regulate target gene expression through reporter gene assays. And the CAD database collected the spatio-temporal information of gene expression driven by CRMs from the REDfly 2.0, literature surveys and their own experiments. In total, we retrieved 1,877 CRMs for 470 genes in *Drosophila melanogaster *from these two databases. We excluded those CRMs with unspecified target genes. Literature-curated TFBSs were mainly gathered from the REDfly database. In REDfly, curated TFBSs were discovered by DNaseI footprinting assays and electrophoresis mobility shift assays (EMSA).

#### Data of chromatin states

Chromatin is composed of DNA molecules and associated proteins. Binding of certain chromatin binding proteins and the post-translational modifications on histones are the major determinant of functionality of regulatory sequences [2,25,26]. By integrating the chromatin protein binding data and some detected histone modifications with computational machine learning models, the global potential principal chromatin types can be revealed [25,26]. Filion *et al*. [25] used 53 selected chromatin components and the hidden Markov model to generate five principal chromatin states. And Kharchenko *et al*. [26] produced a chromatin landscape of nine chromatin states based on 18 histone modifications and the hidden Markov model. We retrieved 8,428 chromatin regions annotated by the chromatin five state model and 20,734 chromatin regions annotated by the nine chromatin combinatorial patterns. By mapping of these global chromatin states to the CRM regions, we can have the potential picture of the transcriptional regulatory functions of the potential CRMs.

#### Data of chromatin accessibility

It is known that gene transcriptional expression is occurring at multiple different time periods throughout development. Hence understanding the temporal manner of CRMs are vital for deciphering the CRM regulatory functionality [27]. Chromatin accessibility have been shown to be related to the spatio-temporal behaviour of gene expression patterns [28]. The genome-scale mapping of the DNaseI hypersensitive sites provides the global chromatin accessibility and the regulatory DNA landscape [28,29]. We adopted the data of chromatin accessible regions which were experimentally identified by the increased DNaseI hypersensitive sites from the work of Thomas *et al*. [28] and Li *et al*. [29]. In their work, temporal behaviours of chromatin accessibility were measured for five different developmental stages (stage 5, 9, 10, 11 and 14) in the Kc167 cell line under 5% FDR control. In total, 105,485 regulator accessible chromatin regions were deposited in *cis*MEP for the five developmental stages in *Drosophila*.

#### Sequence conservation

The primary reason for cross-species sequence conservation is thought to be purifying selection. This results in the fact that sequences that are significantly more similar than would be expected are prone to have critical regulatory functions [30]. CRMs are often built up of highly conserved sequences that are hard-wired into the genomic sequences. Hence analysis of the cross-species sequence conservation can reveal that many of the *cis*-regulatory sequences are actually complex regions representing several enhancers, or else are only enhancer fragments. We adopted the cross-species conservation scores from the work of Siepel *et al*. [30,31]. They have developed a cross-species multiple sequence alignment tool called phastCons, which is based on a two-phase phylogenetic hidden Markov model, to identify conserved elements in multiple-aligned sequences. From their trained and calibrated phylo-HMM model, phastCons generates base-by-base conservation scores for cross-species sequences. The conservation score represents the probability that the sequence is in a conserved element under the trained phylo-HMM model. For *Drosophila*, the conservation scores generated by phastCons were the sequence conservation measurement for the genomes of *Drosophila melanogaster *and 12 other fly species.

#### Experimentally identified promoters

Core promoters are critical DNA regions for gene regulation and transcription initiation. Some CRMs function as gene promoters [3]. Hoskin *et al*. [4] have identified a high resolution map of sequences of promoter regions by three high-resolution experiments: cap analysis of gene expression (CAGE) tags, RNA ligase mediated rapid amplification of cDNA ends (RLM-RACE) reads and cap-trapped expressed sequence tags (ESTs). The promoters are grouped into three categories: validated (V), supported (S) and RACE-only (R). The V group contains promoters defined by two or more experimental data. The S group includes promoters with a CAGE peak or at least three RACE reads. We retrieved 12,454 promoters for 8,008 genes from their work and mapped them to the potential CRM regions as the promoter-type regulatory sequences.

#### Polycomb group binding data

Polycomb group (PcG) proteins are those regulatory proteins repressing the transcription of particular target genes. PcG repression of genes requires specific *cis*-regulatory sequences, forming the silencer CRMs or polycomb response elements (PREs) [32]. Many PcG target genes tend to bear both repression-associated protein binding regions and H3K4me3 [33]. We retrieved the PcG target regions from the work of Schwartz *et al*. [9]. Two types of PcG target regions were classified: Class I regions contain the regions with binding signals of PC protein, E(Z) protein and H3K27me3; Class II regions consist of regions with weaker PC binding and H3K27me3 signals, but without detectable E(Z) signals. 338 class I PcG target regions were collected and 116 class II PcG target regions were gathered for use in this database.

#### Data of annotated regulatory elements

The comprehensive annotation of the regulatory elements in chromatin provides possible functionality of the DNA regulatory sequences in CRMs. In the work of Nègre *et al*. [5], they produced a regulatory map of the *Drosophila melanogaster *genome on the basis of chromatin features, histone deacetylases and site-specific transcription factor binding signals produced by ChIP-chip, ChIP-sequencing or RNA-sequencing techniques across different stages of embryonic, larval, pupal and adult development. The data retrieved and used in *cis*MEP are as the following: 9,058 promoters predicted based on the co-occurrence of H3K4me3 and RNA polymerase II in embryos; 81 enhancers predicted by the binding locations of CREB binding protein (CBP) and RNA polymerase II; 4,774 Class I insulators identified by the binding of CTCF/CP190/BEAF-32 proteins and 2,911 Class II insulators identified by the binding of SU(HW) protein; 537 PREs associated with histone deacetylases (HDACs). These predicted *cis*-regulatory maps provide insight into CRM functions.

#### Histone variant nucleosome data

Eukaryotic DNA is packaged to form nucleosome particles [34]. The location of nucleosomes and the replacement of histones in nucleosomes play vital roles in gene transcriptional regulation and epigenetic inheritance [35]. We gathered two genome-wide detected nucleosome replacement data, the H3.3 replacement and the H2A.Z (also known as H2Av) map, to depict the nucleosome organization in the *Drosophila *genome. H3.3 are known to be related to abundant RNA polymerase II and methylated H3K4 [36]. H2A.Z were reported to be linked to an open and uniform chromatin architecture at promoter regions [37]. In *cis*MEP, we retrieved 617,304 regions with H2A.Z replacement reported by the ChIP-chip technique and 415,119 regions reported to contain the bulk nucleosome (DNA sequences contain any combinations of H2A and H2A.Z) from the work of Mavrich *et al*. [37]. For H3.3 replacement patterns, the original biotin pull-down ratios (including H3.3/wild type and H3.3/H3) were adopted from the work of Mito *et al*. [36] and were mapped to the chromosomal locations of the potential CRMs.

#### Data related to the RNA polymerase II activity

RNA polymerase II is the main molecule responsible for gene transcription. The activity of this polymerase suggests active gene transcription [8]. Hence we collected two types of data representing the RNA polymerase II activity: the regions with RNA polymerase II binding signals and the pausing sites of RNA polymerase II. 9,126 RNA polymerase II binding regions reported by ChIP-sequencing were adopted from the modENCODE project [8] and 3,729 RNA polymerase II pausing regions were collected from the work of Mavrich *et al*. [37].

#### Data identifying histone modifications and chromatin binding proteins

Post-translational modifications of nucleosomal core histones play critical roles in altering chromatin structure and creating target sites for proteins acting on chromatin, thereby regulating gene transcriptional expression [11]. Chromatin binding proteins may function as histone-modifying enzymes related to certain activator or repressor recruitment modifications. Or they may act as the chromatin structure remodelling regulators [7]. In *cis*MEP, we collected 40 different datasets providing the histone modification regions or chromatin protein binding target regions from the work of Kellner *et al*. [38] and the modENCODE project [8]. The .bed files of histone modification data or chromatin protein binding regions identified by the ChIP-chip or the ChIP-sequencing technologies were mapped to the CRM locations. We further categorized the histone modifications and chromatin binding proteins by their potential functionality according to the literature. Details of the functional classification can be seen in Additional File 1.

### Confidence measures for the deposited potential CRMs

In the near future, it will become easier to conduct larger-scale transgenic experiments in Drosophila. Hence it would be desirable to rank the CRMs so that experimentalists can prioritize constructs for transgenic experiments. We have provided two confidence measures to rank the deposited potential CRMs in cisMEP. The first confidence measure is the histone mark confidence score. The histone mark confidence score is calculated by taking the arithmetic mean of the normalized binding scores (the M-values calculated in the ChIP-chip data analysis [8]) of the histone marks overlapping with the CRM. The higher the histone mark confidence score is, the stronger signals of the histone marks are observed. The second confidence measure is the conservation confidence score. This is provided by the phastCons score [30,31] that specifies the sequence conservation among different Drosophila species. Highly conserved CRMs are considered to be more confident potential regulatory elements.

### Implementation of the web service of *cis*MEP

The database is constructed using the PHP language with the CodeIgniter MVC framework. Epigenetic profiling data was deposited through MySQL. Charts displaying the epigenetic profiles for CRMs were produced by the JQuery tool Jqplot.

## Utility and discussion

### Database interface

The *cis*MEP database provides three basic functions for users to select potential CRMs of interest and to view the epigenetic profiles for deciphering potential functionality of the chosen one: (I) search mode, searching for potential CRMs of specific genes or querying potential CRMs with specified annotated functions; (II) browse mode, browsing the database for the computationally identified CRMs deposited in this database; (III) download feature, downloading the plain text file of epigenetic profiles for CRMs. The *cis*MEP web interface provides all entries to these functions.

In the search mode, users can key in the gene of interest to find all potential CRMs related to this gene. Alternatively users can also select the annotated functions defined by the region classification of Nègre *et al*. [5] and Müller *et al*. [32] or specify the intended histone modification combination to find the deposited potential CRMs with the specified epigenetic features. Users can further set the confidence filters of the histone mark confidence and conservation confidence to filter out only those potential CRMs above certain confident probability scores. In the search result page, the computationally identified CRMs satisfying the specified constrains will be listed in a table. In the table, the number of algorithms that specify this sequence as a potential CRM, the number of literature-curated TFBSs residing in the potential CRM region, the number of CRM literature supports for the regulatory region, the histone mark confidence and the conservation confidence for the potential CRM are listed. *cis*MEP also provides the browse mode for users to browse all the deposited CRM prediction results in this database. In the browse mode, users can browse the CRM predictions on different chromosomes or genes. Whether through the search mode or the browse mode, the user can now click the link of "detail" to select the CRM of interest to view the details of the functionally categorized epigenetic profiles for this regulatory sequence.

**Figure 3:**
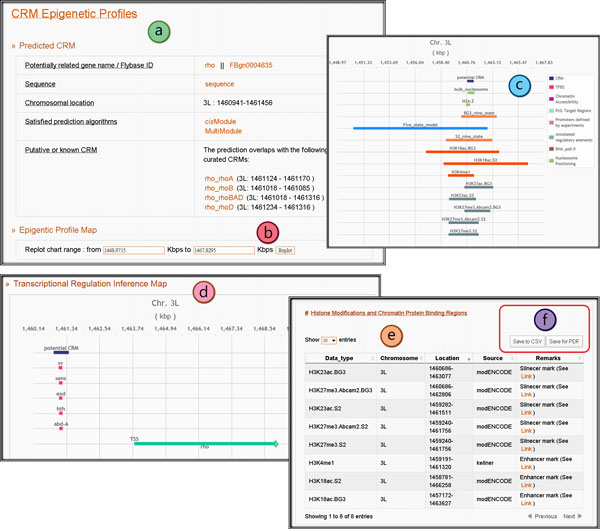
**The CRM epigenetic profile page**. (a) The summary table for the selected potential CRM. (b) The function to zoom in and zoom out the epigenetic profile chart. (c) The epigenetic profile chart for the selected CRM. Epigenetic profiles are rendered as bar tracks if available. (d) The transcriptional regulation inference map for the selected CRM. In this map, TFBSs and gene annotation related to this predicted CRM are shown for transcriptional regulation mechanism inference. (e) The tables of the detailed epigenetic data for the selected CRM. (f) The download function. Tables provided in *cis*MEP can be downloaded as the .csv format or the .pdf form by the users. In this figure, only parts of the data are shown due to space limitations.

In the detail page, users can assess the prediction confidence and decipher possible transcriptional regulatory functions for the chosen CRM. *cis*MEP summarizes the details of the selected potential CRM. The summary elucidates the screening algorithms which this potential CRM satisfied as well as whether it is a putative or known CRM based on literature (Figure 3-a). Next a figure, called the epigenetic profile map, consisting of the genome tracks of the CRM, its functional regions and its epigenetic profiles is provided (Figure 3-c). In the epigenetic profile map, nine types of epigenetic data as described in the Content Section are rendered as horizontal tracks (except for the ratio plots of H3.3 replacements and the genomic distribution of conservation scores) if available. Users can also re-render the axis of the chromosome coordination to zoom in and zoom out the epigenetic tracks of the given CRM (Figure 3-b). Detailed descriptions of specific epigenetic tracks are provided as tool-tips when the users point to them. Below the epigenetic profile plot, we provide the "Transcriptional Regulation Inference Map" that helps visualize the orientations and spacings of TFBSs within the CRMs and helps demonstrate the related gene annotation to this potential CRM and the TFBSs (Figure 3-d). From this map, users can infer the possible transcriptional regulation mechanisms from these TFBS and gene locations. For the raw data of these epigenetic tracks, users can refer to the tables below the chart (Figure 3-e). All tables presented in *cis*MEP can be downloaded easily as the .csv format or as the .pdf format (Figure 3-f).

### Issues related to *cis*MEP

*cis*MEP collects different types of epigenetic data for assessing the confidence of the potential CRMs and deciphering the possible CRM functionality. Yet this type of data integration may result in a potential systematic bias. In systems biology, a cellular system is perturbed and measured by high-throughput technologies. Thus an understanding of the system is based on the integration of different high-throughput assays [39]. But this type of data integration often suffers from the system bias caused by the fact that different high-throughput data were performed on different cell states and cell types [40,41]. Since this type of systematic bias is still unavoidable [40], some critical points are worthy of notice when using the epigenetic profiles for deciphering CRM functionality.

Correct temporal and spatial control of gene expression is crucial in different developmental stages in metazoans. *cis*MEP collects the chromatin accessibility data for different developmental stages and hence provides the temporal behaviour of CRMs. While genome-wide epigenetic data are still not available for all possible combinations of cell types and stages, in *cis*MEP, we have marked out the developmental stages and the cell types for which the high-throughput experiments were performed. Hence in deciphering the CRM functionality, users can also take care of the different cell types for data integration in order not to run the risk of deducing the incorrect inferences.

Besides the potential bias that may result in data integration, due to the nature of high-throughput data, there are still inherent false positives and false negatives in the high-throughput experiment results [39,40], causing the incompleteness in the experimental data support. This insufficiency will be overcome when more and more experiment data are generated. While these data are still not totally complete now, updated version of the experimental validated promoters will be published [4,8] and we will keep *cis*MEP up-to-date with these newly published datasets when more genome-wide data are released.

It is well known that epigenetic marks such as histone variant nucleosomes, histone modifications, and protein binding events are highly dynamic. While looking at these events in a time-sensitive and condition-specific manner is very important for deciphering CRM functionality, the dynamic behaviour of gene expression and CRM regulatory functionality can be considered only in the scope of gene regulatory networks [27]. Construction of the gene regulatory network using the TF-bound CRMs reveals the properties and dynamics of CRM regulation. But challenges remain to be solved in modelling the gene regulatory networks on CRMs [27]. In the current version of *cis*MEP, we aimed to deposit computationally identified CRMs and to provide epigenetic profiles for CRM prediction confidence assessment and potential CRM functionality deciphering. Gene regulatory networks on CRMs will be incorporated in the future updating plan when further advances in gene regulatory network modelling on CRMs are available.

### Case study

To show the application of *cis*MEP, we demonstrate an example describing the epigenetic profiles for one potential CRM residing in the genomic region 3L: 1460941-1461456. This CRM is annotated to be related to the regulation of the gene *rhomboid. rhomboid *is a protein encoding gene in *Drosophila melanogaster *that regulates the fly embryonic development in the ventral neurogenic ectoderm. The CRM regulatory clusters for *rhomboid *gene expression are vital in the formation of patterns of the dorsoventral body axes and the development of the peripheral nervous system [42,43]. We will call this computationally identified CRM as the name of the experimentally verified one, RhoBAD, in the following paragraph for simplicity.

**Figure 4:**
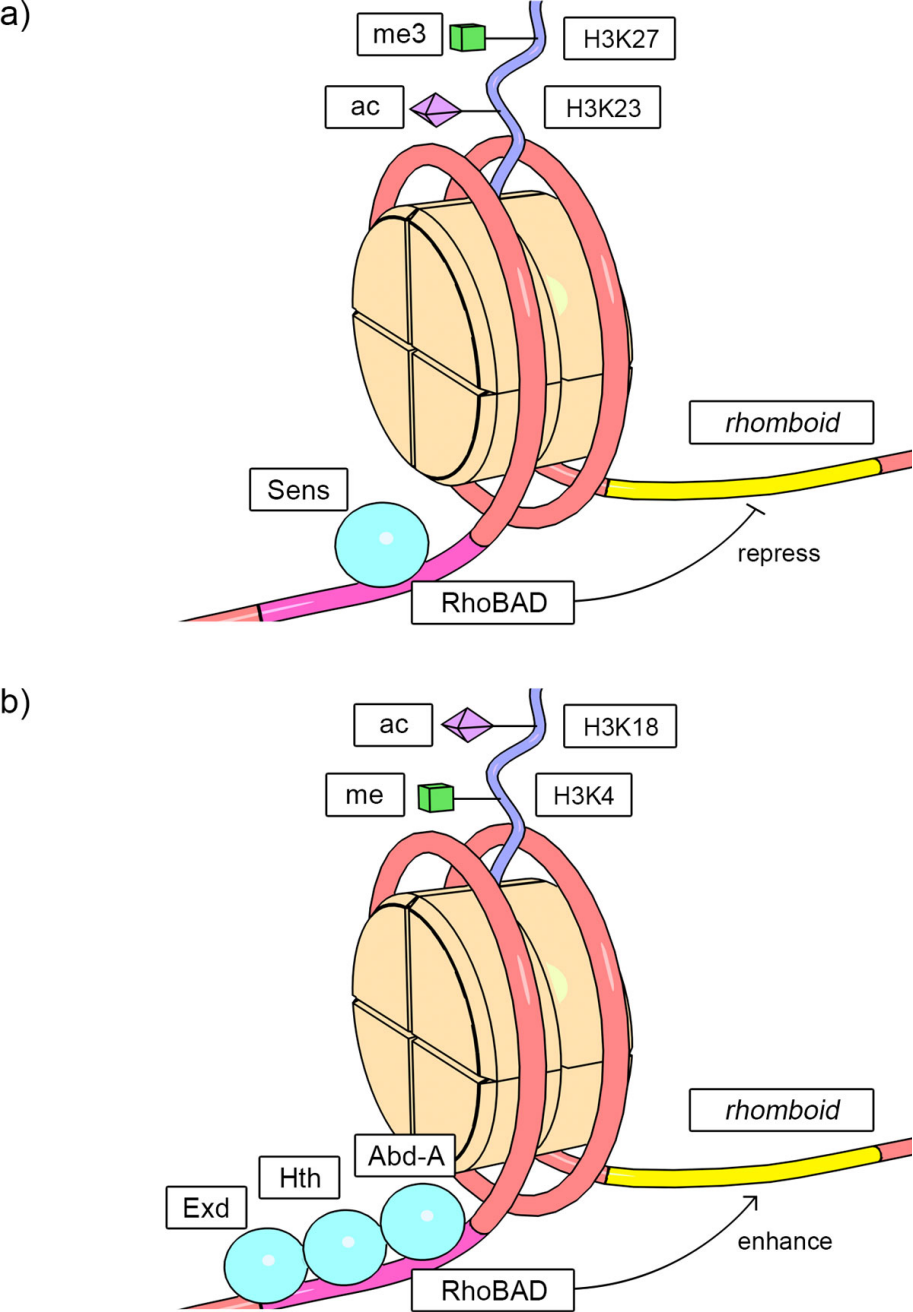
**Simplified sketch plot of the epigenetic profiles and experimental results for RhoBAD**. The epigenetic profiles suggest that the predicted CRM RhoBAD is of high confidence and acts as an enhancer/silencer for the transcription of *rhomboid*. From experimental literature evidence, we know that RhoBAD indeed acts as an enhancer when bound with the Exd/Hth/Abd-A protein complex and acts as a silencer when Sens binds to it. (a) Silencer markers (H3K23ac, H3K27me3). (b) Enhancer markers (H3K4me, H3K18ac).

As shown in the summary table, RhoBAD is predicted by the CRM prediction tools of *cis*Module and MultiModule. And in the epigenetic profiles, we can see that this CRM is enriched in bulk nucleosome (indication of any combination of H2A.Z and H2A) and H2A.Z in embryos, which implies this region is mainly occupied by the histone variant of H2A.Z and suggests the chromatin structure around the promoter region and TSS for actively transcribed genes [37,44]. Enhancer/silencer marks, such as H3K4me1, H3K18ac (marks for enhancers [11,45]), H3K27me3 and H3K23ac (marks for silencers [46,47]) can also be observed in the S2 and BG3 cells. These epigenetic marks imply that this potential regulatory sequence may bear cellular functions and the possible regulatory function of it is gene transcriptional enhancing/silencing. And the conservation score of this predicted CRM reflects the highly conserved tendency of the sequence (conservation probability = 0.56), indicating the potential regulatory functionality of this sequence. From the support of these experimental data, we are quite sure that this CRM prediction is biologically meaningful and may indeed exist and function as the enhancer/silencer CRM for *rhomboid *in living cells. In fact, by the efforts of molecular biologists, four transcription factors- Abdominal-A (Abd-A), Extradenticle (Exd), Homothorax (Hth) and Senseless (Sens)- have been reported to bind to the DNA sequence regions within the RhoBAD CRM [43]. And experimental evidence has revealed the fact that RhoBAD acts as an enhancer when bound with the Exd/Hth/Abd-A protein complex and acts as a silencer when Sens binds to it [43,48]. The sketch of the epigenetic profiles deposited in *cis*MEP and the experimental CRM function verification for RhoBAD are shown in Figure 4. This example shows that the epigenetic profiles gathered in *cis*MEP can help researchers assess the prediction confidence of the deposited potential CRMs, deduce the potential functionality of the computationally identified CRMs and possibly establish the transcriptional regulation mechanism hypotheses of these potential CRMs.

## Conclusions

In this study, we constructed a database called *cis*MEP, which serves to deposit genome-wide systematically screened CRM predictions and to provide the epigenetic profiles as confidence assessment of these potential CRMs. *cis*MEP has an easy-to-use and user-friendly interface for biologists to search and browse CRMs of interest. With TFBSs, epigenetic profiles and chromatin protein binding information, researchers can thus assess the confidence of the potential CRMs and decipher the possible CRM functions. This will facilitate biologists to unravel the regulatory mechanisms of metazoan gene transcription and to figure out the so-called "histone codon" that may possibly reside in transcriptional regulation. Finally, *cis*MEP will be updated on a regular basis to keep in accordance with the latest CRM screening tools, literature-curated CRM data and experimental epigenetic data.

## Availability and requirements

*cis*MEP is available at http://cosbi3.ee.ncku.edu.tw/cisMEP/. JavaScript functioning should be enabled in the user-side browsers and the Adobe Flash Player for specific browsers should also be installed. The web interface of *cis*MEP is fully tested on popular browsers: Microsoft IE9, Google Chrome, Apple Safari and Mozilla Firefox 21. Users are recommended to use these popular browsers for full functionality of *cis*MEP.

## List of abbreviations

CRM, *cis*-regulatory module; TF, transcription factor; ChIP, chromatin immunoprecipitation; TFBS, transcription factor binding site; PcG, polycomb group; PRE, polycomb response element; *cis*MEP, *cis*-regulatory module epigenetic profile database

## Competing interests

The authors declare that they have no competing interests.

## Authors' contributions

THY conceived the research topic. THY and CCW performed the data collection and data processing. THY and PCH constructed the website interface and the database. THY wrote the manuscript and WSW provided essential guidance. All authors have read and approved the final manuscript.

## Declarations

The publication of this manuscript was funded by the Aim for the Top University Project awarded to the National Cheng Kung University.

## Supplementary Material

Additional file 1 **Details of the functional classification of the gathered epigenetic marks**. Additional file 1 contains the table showing the potential regulatory functions of the epigenetic marks. The 'pubmed' column contains the literature supports for the classified regulatory functions. Click here for file

## References

[B1] Davidson EH Erwin DH Gene regulatory networks and the evolution of animal body plans Science 2006 311 796 800 10.1126/science.1113832 16469913

[B2] Jeziorska DM Jordan KW Vance KW A systems biology approach to understanding *cis*-regulatory module function Semin Cell Dev Biol 2009 20 Elsevier 856 862 10.1016/j.semcdb.2009.07.007 19660565

[B3] Hardison RC Taylor J Genomic approaches towards finding cis-regulatory modules in animals Nat Rev Genet 2012 13 469 483 10.1038/nrg3242 22705667 PMC3541939

[B4] Hoskins RA Landolin JM Brown JB Sandler JE Takahashi H Lassmann T Yu C Booth BW Zhang D Wan KH Genome-wide analysis of promoter architecture in Drosophila melanogaster Genome Res 2011 21 182 192 10.1101/gr.112466.110 21177961 PMC3032922

[B5] Nègre N Brown CD Ma L Bristow CA Miller SW Wagner U Kheradpour P Eaton ML Loriaux P Sealfon R A *cis*-regulatory map of the *Drosophila *genome Nature 2011 471 527 531 10.1038/nature09990 21430782 PMC3179250

[B6] Nègre N Brown CD Shah PK Kheradpour P Morrison CA Henikoff JG Feng X Ahmad K Russell S White RA A comprehensive map of insulator elements for the *Drosophila *genome PLoS Genet 2010 6 e1000814 10.1371/journal.pgen.1000814 20084099 PMC2797089

[B7] Kouzarides T Chromatin modifications and their function Cell 2007 128 693 705 10.1016/j.cell.2007.02.005 17320507

[B8] Roy S Ernst J Kharchenko PV Kheradpour P Negre N Eaton ML Landolin JM Bristow CA Ma L Lin MF Identification of functional elements and regulatory circuits by *Drosophila *modENCODE Science 2010 330 1787 1797 21177974 10.1126/science.1198374 PMC3192495

[B9] Schwartz YB Kahn TG Stenberg P Ohno K Bourgon R Pirrotta V Alternative epigenetic chromatin states of polycomb target genes PLoS Genet 2010 6 e1000805 10.1371/journal.pgen.1000805 20062800 PMC2799325

[B10] Van Leeuwen F van Steensel B Histone modifications: from genome-wide maps to functional insights Genome Biol 2005 6 113 10.1186/gb-2005-6-6-113 15960810 PMC1175962

[B11] Boros IM Histone modification in *Drosophila* Brief Funct Genomics 2012 11 319 331 10.1093/bfgp/els029 22806479

[B12] Gallo SM Gerrard DT Miner D Simich M Des Soye B Bergman CM Halfon MS REDfly v3. 0: toward a comprehensive database of transcriptional regulatory elements in *Drosophila* Nucleic Acids Res 2011 39 suppl 1 D118 D123 20965965 10.1093/nar/gkq999 PMC3013816

[B13] Su J Teichmann SA Down TA Assessing computational methods of *cis*-regulatory module prediction PLoS Comput Biol 2010 6 e1001020 10.1371/journal.pcbi.1001020 21152003 PMC2996316

[B14] Sinha S He X MORPH: probabilistic alignment combined with hidden Markov models of *cis*-regulatory modules PLoS Comput Biol 2007 3 e216 10.1371/journal.pcbi.0030216 17997594 PMC2065892

[B15] Bailey TL Noble WS Searching for statistically significant regulatory modules Bioinformatics 2003 19 suppl 2 ii16 ii25 14534166 10.1093/bioinformatics/btg1054

[B16] Frith MC Li MC Weng Z Cluster-Buster: Finding dense clusters of motifs in DNA sequences Nucleic Acids Res 2003 31 3666 3668 10.1093/nar/gkg540 12824389 PMC168947

[B17] Zhou Q Wong WH CisModule: de novo discovery of *cis*-regulatory modules by hierarchical mixture modeling Proc Natl Acad Sci USA 2004 101 12114 12119 10.1073/pnas.0402858101 15297614 PMC514443

[B18] Pierstorff N Bergman CM Wiehe T Identifying *cis*-regulatory modules by combining comparative and compositional analysis of DNA Bioinformatics 2006 22 2858 2864 10.1093/bioinformatics/btl499 17032682

[B19] Zhou Q Wong WH Coupling hidden Markov models for the discovery of *cis*-regulatory modules in multiple species Ann Appl Stat 2007 1 36 65 10.1214/07-AOAS103

[B20] Washington NL Stinson E Perry MD Ruzanov P Contrino S Smith R Zha Z Lyne R Carr A Lloyd P The modENCODE Data Coordination Center: lessons in harvesting comprehensive experimental details Database J Biol Databases Curation 2011 2011 10.1093/database/bar023 PMC3170170 21856757

[B21] Marygold SJ Leyland PC Seal RL Goodman JL Thurmond J Strelets VB Wilson RJ FlyBase: improvements to the bibliography Nucleic Acids Res 2013 41 D751 D757 10.1093/nar/gks1024 23125371 PMC3531214

[B22] Bray N Pachter L MAVID: constrained ancestral alignment of multiple sequences Genome Res 2004 14 693 699 10.1101/gr.1960404 15060012 PMC383315

[B23] Mathelier A Zhao X Zhang AW Parcy F Worsley-Hunt R Arenillas DJ Buchman S Chen C Chou A Ienasescu H JASPAR 2014: an extensively expanded and updated open-access database of transcription factor binding profiles Nucleic Acids Res 2014 42 D142 D147 10.1093/nar/gkt997 24194598 PMC3965086

[B24] Zinzen RP Girardot C Gagneur J Braun M Furlong EE Combinatorial binding predicts spatio-temporal *cis*-regulatory activity Nature 2009 462 65 70 10.1038/nature08531 19890324

[B25] Filion GJ van Bemmel JG Braunschweig U Talhout W Kind J Ward LD Brugman W de Castro IJ Kerkhoven RM Bussemaker HJ Systematic Protein Location Mapping Reveals Five Principal Chromatin Types in *Drosophila *Cells Cell 2010 143 212 224 10.1016/j.cell.2010.09.009 20888037 PMC3119929

[B26] Kharchenko PV Alekseyenko AA Schwartz YB Minoda A Riddle NC Ernst J Sabo PJ Larschan E Gorchakov AA Gu T Comprehensive analysis of the chromatin landscape in *Drosophila *melanogaster Nature 2010 471 480 485 21179089 10.1038/nature09725 PMC3109908

[B27] Wilczynski B Furlong EE Challenges for modeling global gene regulatory networks during development: Insights from *Drosophila* Dev Biol 2010 340 161 169 10.1016/j.ydbio.2009.10.032 19874814

[B28] Thomas S Li XY Sabo PJ Sandstrom R Thurman RE Canfield TK Giste E Fisher W Hammonds A Celniker SE Dynamic reprogramming of chromatin accessibility during *Drosophila *embryo development Genome Biol 2011 12 R43 10.1186/gb-2011-12-5-r43 21569360 PMC3219966

[B29] Li X-Y Thomas S Sabo PJ Eisen MB Stamatoyannopoulos JA Biggin MD The role of chromatin accessibility in directing the widespread, overlapping patterns of *Drosophila *transcription factor binding Genome Biol 2011 12 R34 10.1186/gb-2011-12-4-r34 21473766 PMC3218860

[B30] Siepel A Bejerano G Pedersen JS Hinrichs AS Hou M Rosenbloom K Clawson H Spieth J Hillier LW Richards S Evolutionarily conserved elements in vertebrate, insect, worm, and yeast genomes Genome Res 2005 15 1034 1050 10.1101/gr.3715005 16024819 PMC1182216

[B31] Siepel A Haussler D Phylogenetic hidden Markov models Stat Methods Mol Evol 2005 Springer 325 351

[B32] Müller J Kassis JA Polycomb response elements and targeting of Polycomb group proteins in *Drosophila* Curr Opin Genet Dev 2006 16 476 484 10.1016/j.gde.2006.08.005 16914306

[B33] Bernstein BE Mikkelsen TS Xie X Kamal M Huebert DJ Cuff J Fry B Meissner A Wernig M Plath K A bivalent chromatin structure marks key developmental genes in embryonic stem cells Cell 2006 125 315 326 10.1016/j.cell.2006.02.041 16630819

[B34] Wolffe A Chromatin: Structure and Function 1998 Access Online via Elsevier

[B35] Yuan G-C Liu Y-J Dion MF Slack MD Wu LF Altschuler SJ Rando OJ Genome-scale identification of nucleosome positions in *S. cerevisiae* Science 2005 309 626 630 10.1126/science.1112178 15961632

[B36] Mito Y Henikoff JG Henikoff S Genome-scale profiling of histone H3. 3 replacement patterns Nat Genet 2005 37 1090 1097 10.1038/ng1637 16155569

[B37] Mavrich TN Jiang C Ioshikhes IP Li X Venters BJ Zanton SJ Tomsho LP Qi J Glaser RL Schuster SC Nucleosome organization in the *Drosophila *genome Nature 2008 453 358 362 10.1038/nature06929 18408708 PMC2735122

[B38] Kellner WA Ramos E Van Bortle K Takenaka N Corces VG Genome-wide phosphoacetylation of histone H3 at *Drosophila *enhancers and promoters Genome Res 2012 22 1081 1088 10.1101/gr.136929.111 22508764 PMC3371715

[B39] Ideker T Galitski T Hood L A new approach to decoding life: systems biology Annu Rev Genomics Hum Genet 2001 2 343 372 10.1146/annurev.genom.2.1.343 11701654

[B40] Hwang D Rust AG Ramsey S Smith JJ Leslie DM Weston AD De Atauri P Aitchison JD Hood L Siegel AF A data integration methodology for systems biology Proc Natl Acad Sci USA 2005 102 17296 17301 10.1073/pnas.0508647102 16301537 PMC1297682

[B41] Hwang D Smith JJ Leslie DM Weston AD Rust AG Ramsey S de Atauri P Siegel AF Bolouri H Aitchison JD A data integration methodology for systems biology: experimental verification Proc Natl Acad Sci USA 2005 102 17302 17307 10.1073/pnas.0508649102 16301536 PMC1297683

[B42] Reeves N Posakony JW Genetic Programs Activated by Proneural Proteins in the Developing *Drosophila *PNS Dev Cell 2005 8 413 425 10.1016/j.devcel.2005.01.020 15737936

[B43] Li-Kroeger D Witt LM Grimes HL Cook TA Gebelein B Hox and Senseless Antagonism Functions as a Molecular Switch to Regulate EGF Secretion in the *Drosophila *PNS Dev Cell 2008 15 298 308 10.1016/j.devcel.2008.06.001 18694568 PMC2610489

[B44] Guillemette B Bataille AR Gévry N Adam M Blanchette M Robert F Gaudreau L Variant histone H2A. Z is globally localized to the promoters of inactive yeast genes and regulates nucleosome positioning PLoS Biol 2005 3 e384 10.1371/journal.pbio.0030384 16248679 PMC1275524

[B45] Ong CT Corces VG Enhancer function: new insights into the regulation of tissue-specific gene expression Nat Rev Genet 2011 12 283 293 21358745 10.1038/nrg2957 PMC3175006

[B46] Melgar MF Collins FS Sethupathy P Discovery of active enhancers through bidirectional expression of short transcripts Genome Biol 2011 12 R113 10.1186/gb-2011-12-11-r113 22082242 PMC3334599

[B47] Riddle NC Minoda A Kharchenko PV Alekseyenko AA Schwartz YB Tolstorukov MY Gorchakov AA Jaffe JD Kennedy C Linder-Basso D Plasticity in patterns of histone modifications and chromosomal proteins in *Drosophila *heterochromatin Genome Res 2011 21 147 163 10.1101/gr.110098.110 21177972 PMC3032919

[B48] Witt LM Gutzwiller LM Gresser AL Li-Kroeger D Cook TA Gebelein B Atonal, Senseless, and Abdominal-A regulate *rhomboid *enhancer activity in abdominal sensory organ precursors Dev Biol 2010 344 1060 1070 10.1016/j.ydbio.2010.05.011 20478292 PMC2914175

